# Quantitative evaluation of the impact of artificial cell adhesion via DNA hybridization on E-cadherin-mediated cell adhesion

**DOI:** 10.1063/1.5123749

**Published:** 2020-01-27

**Authors:** Shodai Togo, Ken Sato, Ryuzo Kawamura, Naritaka Kobayashi, Makoto Noiri, Seiichiro Nakabayashi, Yuji Teramura, Hiroshi Y. Yoshikawa

**Affiliations:** 1 Department of Chemistry, Saitama University, 338-8570 Saitama, Japan; 2 Division of Strategic Research and Development, Saitama University, 338-8570 Saitama, Japan; 3 Department of Bioengineering, The University of Tokyo, 113-8656 Tokyo, Japan; 4 Department of Immunology, Genetics and Pathology (IGP), Uppsala University, SE-751 85 Uppsala, Sweden

## Abstract

Programmable cell adhesion with DNA hybridization is a promising approach for fabricating various tissue architectures without sophisticated instrumentation. However, little is known about how this artificial interaction influences the binding of cell adhesion proteins, E-cadherin. In this work, we designed a planar and fluid lipid membrane displaying E-cadherin and/or single-strand DNA with well-defined densities. Visualization of cells on membranes by fluorescence and interference microscopy revealed cell adhesion to be a two-step process: artificial adhesion by DNA hybridization within a few minutes followed by biological adhesion via cadherin-cadherin binding within hours. Furthermore, we discovered that DNA hybridization can substantially facilitate E-cadherin-mediated cell adhesion. The promotive effect is probably due to the enforced binding between E-cadherin molecules in geometrical confinement between two membranes. Our *in vitro* model of cell adhesion can potentially be used to design functional synthetic molecules that can regulate cell adhesion via cell adhesion proteins for tissue engineering.

## I. INTRODUCTION

Tissue engineering, which generates functional and complex three-dimensional tissues *in vitro*, has received considerable attention due to its potential applicability for organ transplantation, disease modeling, and high-throughput drug screening.^1–3^ In particular, because of the substantial development of stem cell technologies in recent decades, various organlike architectures have been achieved by self-organization processes, including self-assembly, self-patterning, and self-driven morphogenesis.^4^ However, it is still challenging to reconstitute high-order, complex tissue structures via self-organization alone. To achieve this goal, bioengineering methods such as molecular recognition-based cell assembly^5–7^ and 3D bioprinting^8–10^ have emerged as powerful tools to rationally control cell arrangement within tissue architectures. In particular, the control of cell-cell adhesion by the hybridization of synthetic DNA is a promising method for fabricating a wide variety of architectures (i.e., higher-order multicellular structures) without sophisticated instrumentation.^6,7^ This technique typically utilizes cell membrane modification with single-stranded DNA (ssDNA) that can quickly bind to other cells with complementary sequences. Since an unlimited number of possible coding specificities can be achieved, this DNA technique can potentially provide a high degree of freedom for the construction of tissue architectures with different cell types. However, there is still an open question regarding the influence of artificial cell-cell adhesion via DNA hybridization on the development and maintenance of the resulting tissues. In nature, cells adhere to their surrounding cells and/or extracellular matrix (ECM) via cell adhesion molecules (CAMs).^11^ The role of CAMs includes not only the physical connection between cells and their environments but also the activation of various intracellular signaling pathways upon cell adhesion. For instance, the intracellular domains of E-cadherin, a well-known cell adhesion protein in vertebrates, form a molecular complex with α/β-catenin, vinculin, and actin fibers,^12–14^ which activates intracellular signaling pathways for various cellular functions and regulates many important physiological processes, including embryonic development, tumorigenesis, wound healing, and maintenance of the structural integrity of epithelia.^15–19^ These facts highlight the importance of CAM-mediated cell adhesion in the reconstitution of functional tissues. Thus, to generate functional tissues, it is essential to clarify the influence of the artificially introduced binding (i.e., DNA hybridization) on CAM-mediated cell adhesion, whereas previous studies focused only on demonstrating the fabrication of tissue architectures via DNA hybridization.

**FIG. 1.**
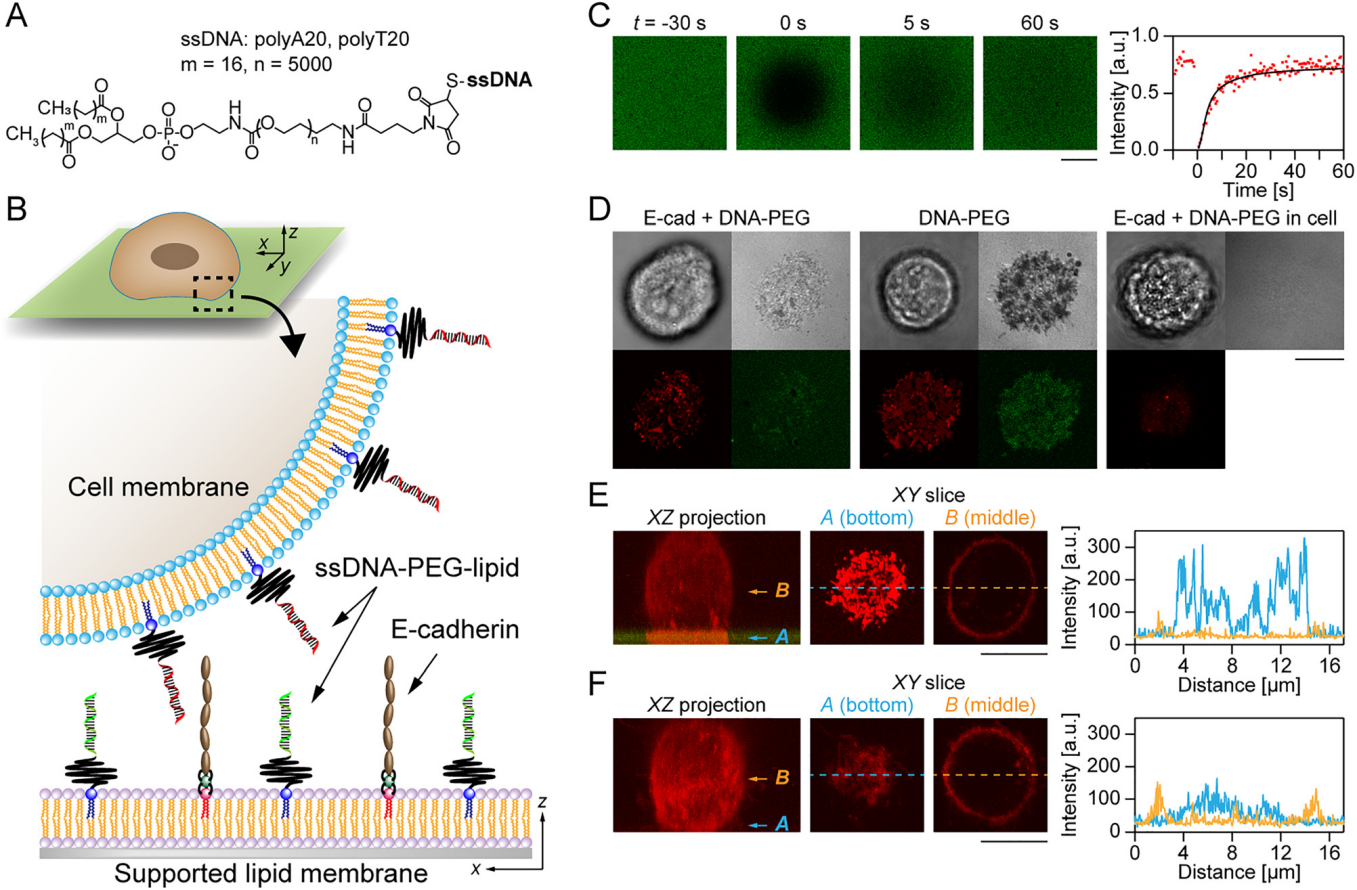
Evaluation of cell adhesion by using living cells and PMs. (a) Molecular structure of a DNA-PEG-lipid that composes ssDNA (polyA/T20), polyethylene glycol (PEG; molecular weight ∼5000), and phospholipid (carbon chain length = 16). (b) Schematic illustration of the *in vitro* model of cell-cell adhesion. A PM was functionalized with E-cadherin and/or DNA-PEG-lipids. Cells (MCF-10A) functionalized with DNA-PEG-lipids having complementary sequences were seeded on the PM. (c) A representative result of FRAP of a PM functionalized with E-cadherin and fluorescein isothiocyanate (FITC)-labeled DNA-PEG-lipids. The right graph shows the time evolution of fluorescence intensity (red) and its best fit (black). (d) Transmission (left top), RICM (right top), and fluorescence images of TAMRA-labeled DNA-PEG-lipids (left bottom) and FITC-labeled DNA-PEG-lipids (right bottom) at *t* = 10 min. (e) and (f) show images of XZ projection (left) and XY slices at a cell-PM interface [(a) corresponds to the blue arrow on XZ projection] and center [(b) corresponds to the orange arrow on XZ projection] of a cell with/without DNA-PEG-lipids, respectively (red: TAMRA-labeled DNA-PEG-lipids on a cell, green: FITC-labeled DNA-PEG-lipid on a PM). The images were acquired at *t* = 5 min. The right-side graph shows the intensity profiles corresponding to the dashed line on the slice images (blue: bottom, orange: middle). It should be noted that brightness and contrast of fluorescence images of (e) and (f) were enhanced to clarify the accumulation of DNA-PEG-lipids at the interface between cells and PMs. Scale bar = 10 *μ*m.

In this study, we evaluated the impact of cell surface modification with DNA-polyethylene glycol (PEG)-lipids, ssDNA, and poly(ethylene glycol)-conjugated phospholipid derivatives^5^ on E-cadherin-mediated cell adhesion [Fig. 1(a)]. For this purpose, we designed a cell-cell adhesion model by seeding living cells onto a planar lipid membrane (PM) displaying E-cadherin and/or DNA-PEG-lipids with well-defined densities [Fig. 1(b)]. Visualization of the cell/PM interfaces by fluorescence and interference microscopy enabled us to distinguish between adhesion sites mediated by E-cadherin and by DNA hybridization. This biophysical approach can provide the first quantitative insights into the mechanism and dynamics of cell-cell adhesion in the presence of artificial and intrinsic interactions.

## II. RESULTS AND DISCUSSION

We prepared a PM of 1-stearoyl-2-oleoyl-*sn*-glycero-3-phosphocholine (SOPC, phase transition temperature ∼6 °C) containing 56 mmol. % 1,2-dioleoyl-*sn*-glycero-3-[(N-(5-amino-1-carboxypentyl)iminodiacetic acid)succinyl] (DGS-NTA), which specifically binds to histidine-tagged E-cadherin via complexation with nickel divalent cations. The PM surface was further functionalized with DNA-PEG-lipids, which can be inserted into the PM via hydrophobic interactions between lipid alkyl chains. To confirm the diffusivity of the functionalized PMs, fluorescence recovery after photobleaching (FRAP) experiments were performed [Figs. 1(c), S1, and S2). Regardless of the surface functionalization, the prepared PMs showed quick fluorescence recovery. The obtained diffusion coefficients, *D*, of DNA-PEG-lipids were 9.3 ± 1.0 *μ*m^2^/s (E-cadherin + DNA-PEG-lipids) and 9.4 ± 2.1 *μ*m^2^/s (DNA-PEG-lipids only), which were comparable to that of fluid SOPC membranes. The fluidic nature of the functionalized membranes enables the precise control of the average lateral distance between ligand molecules with nanometer accuracy. The average distance between E-cadherin molecules, ⟨*d*_cad_⟩, can be estimated with the following equation.^20^

$$\left\langle d_{\mathrm{cad}}\right\rangle =\sqrt{\frac{A_{\mathrm{lipid}}}{x_{\mathrm{DGS}}}}.$$ (1)

With an average area per SOPC (*A*_lipid_) ∼ 65 Å^2^ and a molar fraction of DGS-NTA (Ni) (*x*_DGS_) = 56 mmol. %, the equation yields ⟨*d*_cad_⟩ ∼ 34 nm, which translates to ∼860 molecules/*μ*m^2^. According to the past studies by optical and electron microscopy, the surface density of E-cadherin on living cell membranes was ∼80–500 molecules/*μ*m^2^,^21,22^ which is comparable to that on our PMs. We also estimated the average distance between DNA-PEG-lipid molecules, ⟨*d*_DNA_⟩ ∼4.1 ± 0.1 nm, by fluorescence intensity-based analysis (see Materials and Methods). These results clearly indicate that cofunctionalization with E-cadherin does not hinder the diffusion of DNA-PEG-lipids on the PM.

By using PMs with well-defied ligand densities, we investigated the adhesion behavior of human breast epithelial cells (MCF-10A) functionalized with DNA-PEG-lipids, which possess complementary DNA sequences to those used for the PMs (TAMRA-labeled polyA20 for cells, and FITC-labeled polyT20 for PMs, respectively). MCF-10A endogenously expresses E-cadherin and thus potentially adheres to the E-cadherin-functionalized PM via homophilic interaction, but the surface density of E-cadherin on the cell membrane was initially low because of treatment with trypsin-EDTA for cell harvesting prior to seeding to PM. It should be also noted that the past literature reported ∼6 h as the time required for the complete recovery of E-cadherin expression in the membrane of MCF-10A cells after trypsin-EDTA treatment.^5^ Figure 1(d) shows optical microscopy images of MCF-10A cells on PMs (*t* = 10 min) with three different functionalization conditions: PMs with E-cadherin and DNA-PEG-lipids (PM-cadDNA), DNA-PEG-lipids only (PM-DNA), or E-cadherin only (PM-cad). From the transmission images, the cells showed similar round shapes under all conditions. In contrast, physical contact between cells and PMs [i.e., black regions visualized by reflection interference contrast microscopy (RICM)] was clearly detected when the PM was functionalized with DNA-PEG-lipids. Moreover, confocal fluorescent images showed that the DNA-PEG-lipids on the cell membranes and PMs were colocalized and accumulated at the interface between cells and PMs within 10 min after cell seeding (*t* = 10 min) [Figs. 1(d) left, 1(d) middle, and 1(e)], while no such accumulation occurred for cells on the PMs without DNA-PEG-lipids [Figs. 1(d) right and 1(f)]. The hybridized DNA-PEG-lipids stably remained at the cell/PM interface at *t* = 60 min (Fig. S3). These results indicate that DNA hybridization contributes to the formation of the initial physical contact between cells and PMs.

**FIG. 2.**
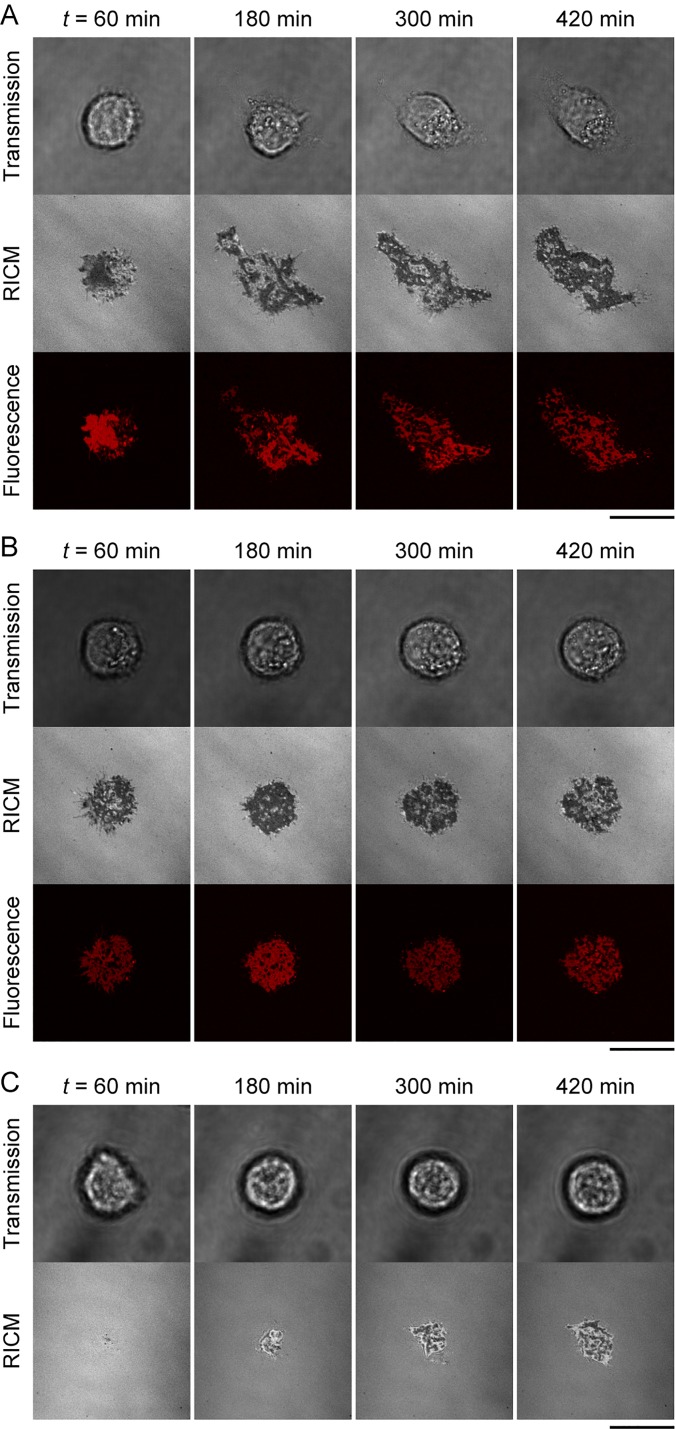
Representative images of adhesion dynamics of cells on (a) PM-cadDNA, (b) PM-DNA, and (c) PM-cad (top: transmission, middle: RICM, bottom: fluorescence from TAMRA-labeled DNA-PEG-lipids on cells). Scale bar = 10 *μ*m.

We investigated the cell adhesion dynamics for longer time scales (*t* ≤ 10 h) to elucidate the impact of DNA-PEG-lipids on the formation of E-cadherin-mediated cell adhesion. Figure 2 shows representative time-course images of cells on PM-cadDNA [Figs. 2(a) and S4(A)], PM-DNA [Figs. 2(b) and S4(B)], and PM-cad [Fig. 2(c)]. In the case of PM-cadDNA, transmission and RICM images show that a cell initially adhered to a PM with a round shape until *t* ∼ 60 min and gradually began to spread with filopodia on the PM from *t* ∼ 180 min. Fluorescence images show that the area of the hybridized DNA-PEG-lipids also expanded as the cell spread. Interestingly, we also observed a region where the cell spread without colocalization of hybridized DNA-PEG-lipids [Fig. 2(a)], whereas the cell remained round in PM-DNA conditions even at *t* = 420 min [Fig. 2(b)]. In addition, the cell on PM-cad showed marginal adhesion at *t* = 60 min and started to slightly increase its adhesion area at *t* ∼ 300 min [Fig. 2(c)]. We found that approximately half of the cells could hardly adhere to the PM-cad, indicating weaker adhesion on PM-cad than on PMs with DNA-PEG-lipids. It should be noted that cells did not adhere to a pure SOPC membrane (no surface functionalization) within 600 min (Fig. S5), indicating that the contact regions visualized by RICM were formed by DNA hybridization and/or cadherin-cadherin binding.

**FIG. 3.**
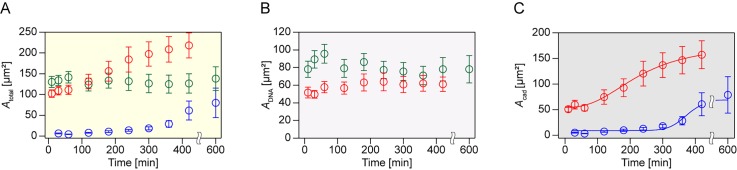
Time evolution of cell adhesion area of (a) *A*_total_, (b) *A*_DNA_, and (c) *A*_cad_ (red: PM-cadDNA, green: PM-DNA, and blue: PM-cad). Solid lines in (c) represent the fitting curve of the empirical Hill equation (red: PM-cadDNA, blue: PM-cad). The plot represents the mean value (±SEM) from n ∼ 50 at each condition.

To quantitatively assess the impact of DNA hybridization on E-cadherin-mediated adhesion, we classified the cell adhesion area into the following parts by image processing techniques: (I) total adhesion area (*A*_total_), (II) DNA hybridization-mediated adhesion area (*A*_DNA_), and (III) E-cadherin–mediated adhesion area (*A*_cad_). Here, *A*_total_ was determined by intensity thresholding of RICM images, which can detect any physical contacts between the PM and cells regardless of the type of attractive interaction. *A*_DNA_ was obtained from the regions where cell/PM contact and the accumulation of DNA-PEG-lipids (determined by intensity thresholding of fluorescence images) overlapped. Then, *A*_cad_ was determined by simply subtracting *A*_DNA_ from *A*_total_ (see Fig. S6 and the Materials and Methods section for a more detailed explanation). Since cell adhesion was mainly formed by DNA hybridization and/or cadherin-cadherin interaction, *A*_cad_ corresponds approximately to the adhesion region mediated by the homophilic interaction between E-cadherin molecules. It should be noted that *A*_total_ is treated as *A*_cad_ in the case of the PM-cad condition since there is no DNA hybridization. Figure 3 shows a summary of *A*_total_, *A*_DNA_, and *A*_cad_ (see Fig. S7 for histograms of each adhesion area). In the initial stage of cell adhesion (*t* ≤ 100 min), *A*_total_ of PM-cadDNA, and *A*_total_ of PM-DNA were both ∼150 *μ*m^2^, approximately 15 times larger than that of PM-cad (∼10 *μ*m^2^) [Figs. 3(a) and S7(A)]. Afterwards, *A*_total_ in the presence of E-cadherin gradually increased and finally reached a plateau (∼250 *μ*m^2^ for PM-cadDNA and ∼100 *μ*m^2^ for PM-cad, respectively) at 600 min, whereas it remained almost constant in the absence of E-cadherin (PM-DNA). *A*_DNA_ was almost constant for both PM-cadDNA (∼60 *μ*m^2^) and PM-DNA (∼80 *μ*m^2^) [Figs. 3(b) and S7(B)]. In contrast, *A*_cad_ of PM-cadDNA and PM-cad showed transitionlike behavior similar to that of their *A*_total_ [Figs. 3(c) and S7(C)]. To evaluate the characteristic time scale of the transition of cell adhesion, *A*_cad_ of PM-cadDNA and PM-cad was fitted with the empirical Hill equation^23^

$$A_{\mathrm{Ecad}}=\alpha +\frac{\beta t^{\gamma }}{{t_{\mathrm{half}}}^{\gamma }+t^{\gamma }},$$ (2)

where *α* and *β* are the constant, *t*_half_ is a half-max modulus, and *γ* is the co-operativity coefficient. The best fit yields a *t*_half_ of 231.2 ± 23.8 min for PM-cadDNA and 373.9 ± 14.9 min for PM-cad (Table I).

**TABLE I.** Fitting coefficients of t_half_ and γ for PM-cadDNA and PM-cad (value ± SD).

	PM-cadDNA	PM-cad
*t*_half_ (min)	231.2 ± 23.8	373.1 ± 15.7
*γ*	2.7 ± 0.5	13.8 ± 6.7

The cell adhesion dynamics results suggest that cell adhesion to PM-cadDNA involves a two-step process: artificial adhesion via DNA hybridization within a few minutes followed by biological adhesion via cadherin-cadherin binding within hours. The underlying mechanism of this two-step adhesion process can be explained by the difference in reaction kinetics between E-cadherin and DNA-PEG-lipids. Regarding the reaction kinetics of E-cadherin, for simplicity, we here assume two-dimensional adhesion between two different PMs. Wu *et al.* calculated the two-dimensional molecular kinetic constant *K_D_*^(^^2D^^)^ (molecules/*μ*m^2^ as unit), which is analogous to the three-dimensional dissociation constant *K_D_* (mol l^−1^),^24^ of E-cadherin trans-dimers to be ∼250 molecules/*μ*m^2^. We found comparable values for the two-dimensional density of E-cadherin on PM (∼860 molecules/*μ*m^2^) and the surface density of E-cadherin on living cell membranes (∼80–500 molecules/*μ*m^2^).^21,22^ Thus, in this density condition, E-cadherin-mediated adhesion between the cell membrane and synthetic PM can be promoted after cell seeding. However, we should also consider that the MCF-10A cells used in this study were treated with trypsin-EDTA prior to seeding, which should decrease the surface density of E-cadherin on the cell membrane during the early time scale. Teramura reported that E-cadherin in the membrane of MCF-10A cells after trypsin-EDTA treatment increases in a sigmoidal manner and requires over 6 h for complete recovery,^5^ which is consistent with the *t*_half_ under PM-cad conditions (∼370 min). This result suggests that the dynamics of cell adhesion on PM-cad was mainly governed by the recovery time scale of E-cadherin expression.

The two-dimensional dissociation constant *K_D_*^(2D)^ of DNA-PEG-lipids (A20/T20 base pairs) can be estimated by *K_D_*^(2D)^ = *hK_D_*, where h is the constrained height. By assuming *h* = 7 nm (20 base pairs ∼6.8 nm) and *K_D_* ∼ 10 pM,^25,26^ one can obtain *K_D_*^(2D)^ ∼4 × 10^−5^ molecules/*μ*m^2^, which is 10^7^ times smaller (larger affinity) than *K_D_*^(2D)^ of E-cadherin. Considering that DNA-PEG-lipids were densely functionalized on the cell membranes and PMs (∼6 × 10^4^ molecules/*μ*m^2^), the adhesion between cells and the PM should be completed by DNA hybridization immediately after cell seeding. Indeed, *A*_DNA_ of both the PM-cadDNA and PM-DNA conditions reached saturation at *t* = 10 min and became almost constant for 600 min. In addition, as revealed by FRAP experiments, DNA-PEG-lipids are highly mobile in both the fluid PM and PM-cadDNA with diffusion constants of ∼9.3 and 9.4 *μ*m^2^/s, respectively. Since cell membranes are also fluid (*D* of non-raft-related fractions on cell membrane > 1 *μ*m^2^/s at 37 °C),^27,28^ DNA-PEG-lipids can undergo free diffusion in both cell membranes and PMs. If we use the obtained diffusion constant in PM (*D* ∼ 9.4 *μ*m^2^/s) and a typical cell diameter (*φ* = 10 *μ*m), the time required for the diffusion of DNA-PEG-lipids on the entire cell surface can be calculated as 4π(*φ*/2)^2^/*D* ∼ 134 s, indicating that DNA-PEG-lipids can travel over the entire cell membrane within a few minutes. The results of Figs. 1(e) and S3(A) clearly showed that the accumulation of DNA-PEG-lipids at the cell/PM interface was completed within 10 min. Such fast diffusion together with the high affinity and surface density of DNA-PEG-lipids should lead to the prompt formation of tight contacts between cells and PM via hybridization prior to E-cadherin dimerization.

Considering the above-mentioned difference in reaction kinetics between E-cadherin and DNA-PEG-lipids, it is comprehensible that cell adhesion to PM-cadDNA involves a two-step process. The large difference in *γ*, the co-operativity coefficient, between PM-cadDNA and PM-cad implies that the dynamics of E-cadherin binding upon cell adhesion is significantly modulated by DNA-PEG-lipids. The obtained half-max moduli, *t*_half_, should represent characteristic time scales for the transition from artificial adhesion via DNA hybridization to biological adhesion via E-cadherin. Interestingly, *t*_half_ for PM-cadDNA was approximately half that for PM-cad, which indicates that E-cadherin-mediated cell adhesion in the presence of DNA-PEG-lipids proceeds faster than without DNA-PEG-lipids. This difference in rate may be explained by the biophysical and/or biochemical roles of E-cadherin binding in cell adhesion. As shown in Fig. 3, cells on PM-cadDNA exhibit a substantial area (∼100 *μ*m^2^) for cell adhesion immediately after cell seeding compared to the case of PM-cad (<10 *μ*m^2^). In addition, the length of the extracellular domains of DNA-PEG-lipid dimers (polyA20/T20 pair ∼20 nm) is short enough to bring E-cadherin within the length scale of the extracellular domains of E-cadherin dimers (∼40 nm).^5,29,30^ Therefore, the larger adhesion area mediated by DNA hybridization should provide a larger field for collision and subsequent dimerization between E-cadherin molecules. Furthermore, this induction of E-cadherin binding by DNA hybridization may accelerate the expression of E-cadherin. Some studies have reported that cadherin-cadherin binding can upregulate the expression of E-cadherin via β-catenin or p120-catenin signaling pathways.^18,19^ Thus, it is plausible that initial contact between cells and PMs mediated by DNA hybridization can promote the binding and subsequent expression of E-cadherin, leading to the faster formation of E-cadherin-mediated cell adhesion on PM-cadDNA than in the absence of DNA-PEG-lipids.

## III. CONCLUSION

In this work, the effect of artificial cell attachment via DNA hybridization on E-cadherin-mediated cell adhesion was elucidated by using an *in vitro* model system. Fluid PMs functionalized with a well-defined molecular density of E-cadherin and DNA-PEG-lipids allowed us to quantify the impact of DNA hybridization and E-cadherin binding on the adhesion between cells and PMs. We found cell adhesion to be a two-step process: physical contact mediated by DNA hybridization within minutes and the subsequent E-cadherin-mediated adhesion in hours. Furthermore, we discovered that DNA hybridization can facilitate E-cadherin-mediated cell adhesion. We note that such adhesion promotion via DNA-PEG-lipids may occur with other members of the cadherin family such as N-cadherin because N-cadherin exhibits a similar autoregulation mechanism.^31^ In addition, the fine-tuned spacing between cell membranes with DNA-PEG lipids should also promote bindings of various cell adhesion molecules. Therefore, we expect that our *in vitro* model system can be potentially used to design molecular structures that can promote cell adhesion via various cell adhesion proteins, which should become a promising approach in constructing highly ordered, biologically functional tissues using synthetic molecules.

## IV. MATERIAL AND METHODS

### A. Cell culture

Human breast epithelial cells (MCF-10A) were obtained from the American Type Culture Collection (USA) and routinely cultured in DMEM/F12 (Dulbecco's Modified Eagle Medium:Nutrient Mixture F-12) medium (Thermo Fisher Scientific, USA) containing 10% horse serum (Thermo Fisher Scientific), 20 ng/ml human epidermal growth factor (hEGF, Pepro Tech, USA), 0.5 *μ*g/ml hydrocortisone (Sigma-Aldrich, USA), 20 *μ*g/ml insulin (Sigma-Aldrich), and 50 unit/ml penicillin-streptomycin (Thermo Fisher Scientific). Cells were maintained in an incubator at 37 °C with 5% CO_2_ supply.

### B. Preparation of small unilamellar vesicle (SUV)

SOPC and DGS-NTA (Ni) lipid powders were purchased from Avanti Polar Lipid (USA) and used without further purification. SOPC or DGS-NTA (Ni) powder was dissolved into chloroform and stored at −20 °C as stock solutions [10 mg/ml for SOPC and 0.1 mg/ml for DGS-NTA (Ni), respectively]. To prepare SUV, 200 *μ*l of SOPC and 1.5 *μ*l of DGS-NTA (Ni) from the stock solutions were mixed in a glass vial, which was cleaned by the modified RCA protocol beforehand.^20,32^ The molar ratio of DGS-NTA (Ni) can be estimated to be 56 mmol. % with respect to SOPC. The chloroform in the mixture was evaporated under gentle nitrogen gas flow to form a dry lipid film and then stored in a vacuum chamber overnight to remove the residual solvent. Next, a dry lipid film was suspended in 4 ml of HEPES-buffered saline (HBS, 10 mM HEPES, 150 mM NaCl, pH 7.4) and incubated for 1 h at 37 °C. The lipid suspensions were then sonicated by using a tip sonicator (Q55, Qsonica, USA) for 60 min. Impurities (mostly fragments of the titanium tip of the sonicator) were precipitated by centrifugation (14 800 rpm for 10 min), and the supernatants were collected as the SUV solution. The final concentration of SOPC in the solution was 0.5 mg/ml. In this study, we also prepared SUV solution containing 0.5 mg/ml SOPC + 0.5 mol. % Texas Red 1,2-dihexadecanoyl-*sn*-glycero-3-phosphoethanolamine (Texas Red-DHPE, Thermo-Fisher Scientific) to confirm the deposition of PMs on glass substrates as well as their diffusivity.

### C. Preparation of PM and its functionalization with E-cadherin and/or DNA-PEG-lipid

Poly(dimethylsiloxane) (PDMS, Sylgard 184, Dow Corning Toray, Japan) and a coverslip (Matsunami Glass, Japan) were used to prepare a chamber for this experiment. A silicone prepolymer mixed with a cross-linker at a ratio of 10:1 (in volume) was baked for more than 3 h at 80 °C. Afterwards, PDMS was hollowed out by a punch with a diameter of 6 mm. The coverslip was cleaned and hydrophilized by the modified RCA protocol, followed by treatment with an UV/Ozone cleaner (ProCleaner Plus, BioForce Nanoscience, USA). Finally, the coverslip was fixed together with the hollowed PDMS, which was also treated with O_2_ plasma, to form the chamber. PMs were prepared by the deposition of 100 *μ*l of SUV solution in the chamber for 30 min at room temperature. After the removal of unbound lipids by washing with HBS, 10 mM NiCl_2_ solution was added to the chamber (final conc. 1 mM) and incubated for 30 min at room temperature. The chamber was again washed with HBS, and 8 *μ*l of 250 *μ*g/ml histidine-tagged E-cadherin (R&D Systems, USA) was added and incubated for 90 min at room temperature, which allows for the uniform functionalization of a planar membrane with E-cadherin via the Ni^2+^ chelation.^20,33^ Thereafter, the chamber was washed again with HBS, and 4 *μ*l of 26 *μ*g/ml FITC-labeled polyT20-PEG-lipid solution, which was synthesized as previously described,^5,34^ was added and incubated for 30 min at 37 °C. Finally, the PM was rinsed with L15 medium (20 mM HEPES buffered, Thermo-Fisher Scientific) to remove excess components. In the case of the FRAP measurement of E-cadherin (Fig. S1), fluorescently labeled E-cadherin was prepared by mixing a 6-Carboxytetramethylrhodamine, Succinimidyl Ester (TAMRA-SE, Thermo-Fisher Scientific) according to the manufacture's protocol. Then the TAMRA-labeled E-cadherin was anchored on a PM with 5.6 mol. % of DGS-NTA under the same procedure as mentioned above.

### D. Modification of the cell membrane with DNA-PEG-lipid

Cultured cells were harvested with trypsin-EDTA similar to past works of tissue engineering with DNA-PEG lipids^5,35^ and then collected by centrifugation for 3 min at 1000 rpm. After removal of the supernatants, the cell pellets were incubated with 30 *μ*l of 260 *μ*g/ml TAMRA-labeled polyA20-PEG-lipid solution for 30 min at 37 °C. Cells were then washed and resuspended in L15 medium and seeded onto the PM in the chamber.

### E. Microscopy

RICM, fluorescence, and transmission images were acquired by using an inverted laser scanning confocal microscope system (A1R+, Nikon, Japan). A 63× oil immersion objective lens equipped with a quarter-wave plate (NA 1.25, Plan Neofluar, Zeiss, Germany) was used. A cross polarizer setup was employed to remove stray light that obscured the signal of interest. The appearance of contrast in an RICM image depends on the distance between the substrate and the object, *h*, as described by the following equation:^36^

$$\frac{2I-\left(I_{\text{max}}+I_{\text{min}}\right)}{-\left(I_{\text{max}}-{\;I}_{\text{min}}\right)}=\text{cos}\left(\frac{4\pi n}{\lambda }h\right).$$ (3)

Here, *I* is the measured intensity, *I*_max_ and *I*_min_ are the maximum and minimum intensity, *n* is the reflective index of the medium (1.33), and *λ* is the wavelength of an incident laser (405 nm). Thus, the height profile of the cell interface can be reconstructed from RICM images using Eq. (3). In fluorescence imaging, lasers with wavelengths of 488 nm and 561 nm were used for FITC and TAMRA, respectively. The transmission images were acquired simultaneously with the fluorescence images.

### F. Image analysis

ImageJ software (NIH) was used for image analysis. The total adhesion area *A*_total_ was determined from the RICM images. First, the RICM image was processed with a “Subtract Background” function, and then, the contour of the cell adhesion area was clipped. Then, the highest and lowest intensities in the clipped image were determined, and the pixels described by the following equation were extracted by thresholding:

$$I_{\text{min}}\leq I\leq \chi \cdot I_{\text{max}}.$$ (4)

Here, *χ* is the threshold value. In this study, *χ* was fixed at 0.65, corresponding to *h* ∼45 nm in Eq. (3). According to previous reports, the extracellular domains of hybridized DNA-PEG-lipids (polyA20/T20 pair) and E-cadherin dimers are ∼20 nm and ∼40 nm, respectively.^5,29,30^ Thus, we can identify the cell adhesion area mediated by either DNA-PEG-lipid or E-cadherin from the RICM images. We defined the total area of extracted pixels as *A*_total_. To calculate the DNA hybridization-mediated cell adhesion area (*A*_DNA_), the obtained fluorescence image was treated with thresholding using the Iso-Data method. Then, the binary RICM and fluorescence images were processed with the “AND” command of the “Image Calculator” function, which gives *A*_DNA_ as an overlapping area. *A*_cad_ was determined by simply subtracting *A*_DNA_ from *A*_total_ using the “SUBTRACT” command of the “Image Calculator” function.

### G. FRAP measurement

FRAP experiments were carried out by using the same laser scanning confocal microscope mentioned in the microscopy section at 37 °C. We used a 100× oil immersion objective lens (NA 1.49, Apo SR TIRF, Nikon). A circular region of the PM (*r* = 10 *μ*m) was bleached with femtosecond laser pulses (800 nm, 2920 mW, 80 MHz, Chameleon Vision-S, Coherent, USA). Fluorescence images were simultaneously recorded at 2 frames per second by using a 488 nm laser line. To eliminate the effect of natural photobleaching, the time course fluorescence intensity of the center of the bleached region was normalized to the intensity of a nonbleached region (typically bottom left of the image). Plotted fluorescence curves were then fitted with the following equation, which is based on FRAP theory.^37^

$$f\left(t\right)=a+b\cdot \exp\left(\frac{-2\tau_{D}}{t}\right)\left\{I_{0}\left(\frac{2\tau_{D}}{t}\right)+I_{1}\left(\frac{2\tau_{D}}{t}\right)\right\}.$$ (5)

Here, *f* is the normalized fluorescence intensity, *a* and *b* are constants, *t* is time, *I*_0_ and *I*_1_ are modified Bessel functions, and *τ*_D_ is the fluorescence recovery time. We determined the diffusion coefficient *D* using $\tau_{D}=w^{2}/4D$, where *w* is the radius of the bleached region. The fitting was carried out by using Igor Pro 7 software (WaveMetrics, USA). We obtained the mean values of *D* from more than four samples for each PM-cadDNA, PM-DNA, PM-cad, and Texas red-DHPE-labeled SOPC membrane condition.

### H. Determination of the mean distance between DNA-PEG-lipids

The mean distance between DNA-PEG-lipids within PMs was calculated from the concentration of unbound DNA-PEG-lipids. First, fluorescence spectra of HBS solutions containing FITC-labeled DNA-PEG-lipids with known concentrations (0.5–2 *μ*g/ml) were obtained by using a fluorescence spectrometer (FP-6300, JASCO, Japan) [Fig. S8(A)]. The excitation wavelength was 495 nm. Then, a calibration curve was obtained by plotting the fluorescence intensities at 520 nm as a function of the concentration of DNA-PEG-lipids [Fig. S8(B)]. Next, PMs with E-cadherin were prepared in a PDMS chamber with a hollow diameter of 20 mm, which is larger than those used in the cell experiments. The PM was incubated with 1 ml of a solution containing 1.5 *μ*g/ml FITC-labeled DNA-PEG-lipids for 30 min at 37 °C. The solution was then diluted to 2 ml. After gentle pipetting, 1 ml of the solution was collected, and the fluorescence spectrum was measured by using a fluorescence spectrometer. The concentration of unbound DNA-PEG-lipids was calculated from the calibration curve. Finally, by taking the molecular weight of DNA-PEG-lipids (5000) and the known chamber area, a mean distance between DNA-PEG-lipids ⟨*d*_DNA_⟩ could be determined. We conducted three independent experiments to determine the mean distance.

### I. Ethics approval

Ethics approval is not required to carry out this work.

## SUPPLEMENTARY MATERIAL

See the supplementary material for FRAP results of PMs functionalized with TAMRA-labeled E-cadherins, FRAP results of PMs functionalized with FITC-labeled DNA-PEG-lipids or Texas red-DHPE (no DNA-PEG-lipids), confocal images of a cell with or without DNA-PEG-lipids at *t* = 60 min, lower magnification images of the adhesion dynamics of cells on PM-cadDNA and PM-DNA, images of the adhesion dynamics of cells on a pure lipid membrane, scheme of cell adhesion area analysis conducted using ImageJ software, time-course histograms of adhesion area, and quantification of DNA-PEG-lipids on PMs.
